# Rapid and Sensitive Detection of *Shigella* spp. and *Salmonella* spp. by Multiple Endonuclease Restriction Real-Time Loop-Mediated Isothermal Amplification Technique

**DOI:** 10.3389/fmicb.2015.01400

**Published:** 2015-12-14

**Authors:** Yi Wang, Yan Wang, Lijuan Luo, Dongxin Liu, Xia Luo, Yanmei Xu, Shoukui Hu, Lina Niu, Jianguo Xu, Changyun Ye

**Affiliations:** ^1^State Key Laboratory of Infectious Disease Prevention and Control, Collaborative Innovation Center for Diagnosis and Treatment of Infectious Diseases, Chinese Center for Disease Control and Prevention, National Institute for Communicable Disease Control and PreventionBeijing, China; ^2^Pathogenic Biology Institute, University of South ChinaHengyang, China; ^3^School of Tropical and Laboratory Medicine, Hainan Medical UniversityHaikou, China

**Keywords:** *Shigella* spp., *Salmonella* spp., MERT-LAMP, LAMP, LoD

## Abstract

*Shigella* and *Salmonella* are frequently isolated from various food samples and can cause human gastroenteritis. Here, a novel multiple endonuclease restriction real-time loop-mediated isothermal amplification technology (MERT-LAMP) were successfully established and validated for simultaneous detection of *Shigella* strains and *Salmonella* strains in only a single reaction. Two sets of MERT-LAMP primers for 2 kinds of pathogens were designed from *ipaH* gene of *Shigella* spp. and *invA* gene of *Salmonella* spp., respectively. Under the constant condition at 63°C, the positive results were yielded in as short as 12 min with the genomic DNA extracted from the 19 *Shigella* strains and 14 *Salmonella* strains, and the target pathogens present in a sample could be simultaneously identified based on distinct fluorescence curves in real-time format. Accordingly, the multiplex detection assay significantly reduced effort, materials and reagents used, and amplification and differentiation were conducted at the same time, obviating the use of postdetection procedures. The analytical sensitivity of MERT-LAMP was found to be 62.5 and 125 fg DNA/reaction with genomic templates of *Shigella* strains and *Salmonella* strains, which was consist with normal LAMP assay, and at least 10- and 100-fold more sensitive than that of qPCR and conventional PCR approaches. The limit of detection of MERT-LAMP for *Shigella* strains and *Salmonella* strains detection in artificially contaminated milk samples was 5.8 and 6.4 CFU per vessel. In conclusion, the MERT-LAMP methodology described here demonstrated a potential and valuable means for simultaneous screening of *Shigella* and *Salmonella* in a wide variety of samples.

## Introduction

*Shigella* and *Salmonella*, recognized as important human pathogens worldwide, are responsible for food-borne gastroenteritis, which cause bacterial diarrhea represent a huge public health problem (Yang et al., 2007). It was estimated that in the word about 1.8 million cases died from diarrheal illnesses, a large proportion of which were attributed to *Shigella* spp. and *Salmonella* spp. (Shao et al., 2011). Previous studies have reported that *Shigella* app. were associated with the majority of cases of bacillary dysentery prevalent in developing nations, and *Salmonella* spp. were the most common cause of food-borne infection outbreaks in almost all countries (Iseki et al., 2007; Lin et al., 2010; Lauri et al., 2011). Therefore, for food industry and public health, rapid, specific and sensitive methodologies to detect the pathogens are continuously required.

The culture-based techniques were used as the gold standard for the detection of *Shigella* spp. and *Salmonella* spp. in various samples, but the conventional procedures required multiple subculture steps, biochemical and serological confirmation, which took about 7 days, and were time-consuming and laborious (Mokhtari et al., 2012; Soria et al., 2013). In recent years, molecular technologies, such as PCR and real-time PCR assays, have been successfully applied to detect *Shigella* spp. and *Salmonella* spp., while these techniques might not be available for resource-poor regions because of the requirement of specialized laboratories, trained personnel, complicated apparatus and expensive reagents (Okamura et al., 2009; Liew et al., 2014; Wang et al., 2015b). In addition, PCR-based technologies obtained poor performance in practical samples, which contained low numbers of bacterial cells, thus, these techniques required an amplification of bacterial numbers by culturing practical samples for 4–8 h (Song et al., 2005). Herein, it is extremely critical to establish a simple, rapid, sensitive, and specific approaches for detection of *Shigella* spp. and *Salmonella* spp. in medical, field and food diagnostic laboratories.

Loop-mediated isothermal amplification (LAMP), as a simple, rapid, highly specific, and sensitive detection methodology, has been employed to detect various pathogens, including parasites, fungi, bacteria, and viruses (Mori and Notomi, 2009; Law et al., 2014). Due to detect a single target sequence, the diagnostic assay has been limited its more flexible and wider applications (Fang et al., 2011; Wang et al., 2014). In order to achieve multiplex LAMP (mLAMP) detection, end point analysis, such as agarose gel and pyrosequencing methods, have been employed to differentiate multiple target sequences, while these strategies required further processing and instrumentation, and did not permit real-time detection (Yang et al., 2007; Liang et al., 2012). More recently, a novel technology presented in our previous study, multiple endonuclease restriction real-time loop-mediated isothermal amplification (MERT-LAMP), overcame the technical difficulties posed by current mLAMP approaches, which was demonstrated to simultaneously detect multiple targets in a single reaction (Wang et al., 2015a). Furthermore, the MERT-LAMP approaches allowed real-time detection in only one isothermal amplification step and the positive results could be obtains in as short as 12 min. As such, the novel MERT-LAMP methodology constitutes a potentially valuable tool for rapid detection of a variety of pathogens in either commercial or field labs.

In present study, our objective is the development and validation of a diagnostic MERT-LAMP-based methodology for simultaneous detection of the *Shigella* spp. and *Salmonella* spp. with high specificity and sensitivity, by targeting *ipaH* (GenBank accession no. M32063) and *invA* (GenBank accession no. NC.003197) genes, respectively. The *ipaH* gene, coding an invasion-associated plasmid antigen, was present in multiple copies in both the chromosomes and the plasmids of all *Shigella* species, which could be selected as mark gene for detection of all *Shigella* strains (Gaudio et al., 1997; Vu et al., 2004). The *Salmonella* invasion gene (*invA*), has been demonstrated to exist in all *Salmonella* strains but not in non- *Salmonella* bacteria, thus, the feature allowed to establish *invA*-based molecular detection methods for diagnosis of all *Salmonella* stains (Galan et al., 1992; Vantarakis et al., 2000; Hara-Kudo et al., 2005).

## Materials and methods

### Design of the MERT-LAMP primers

**Figure 1:**
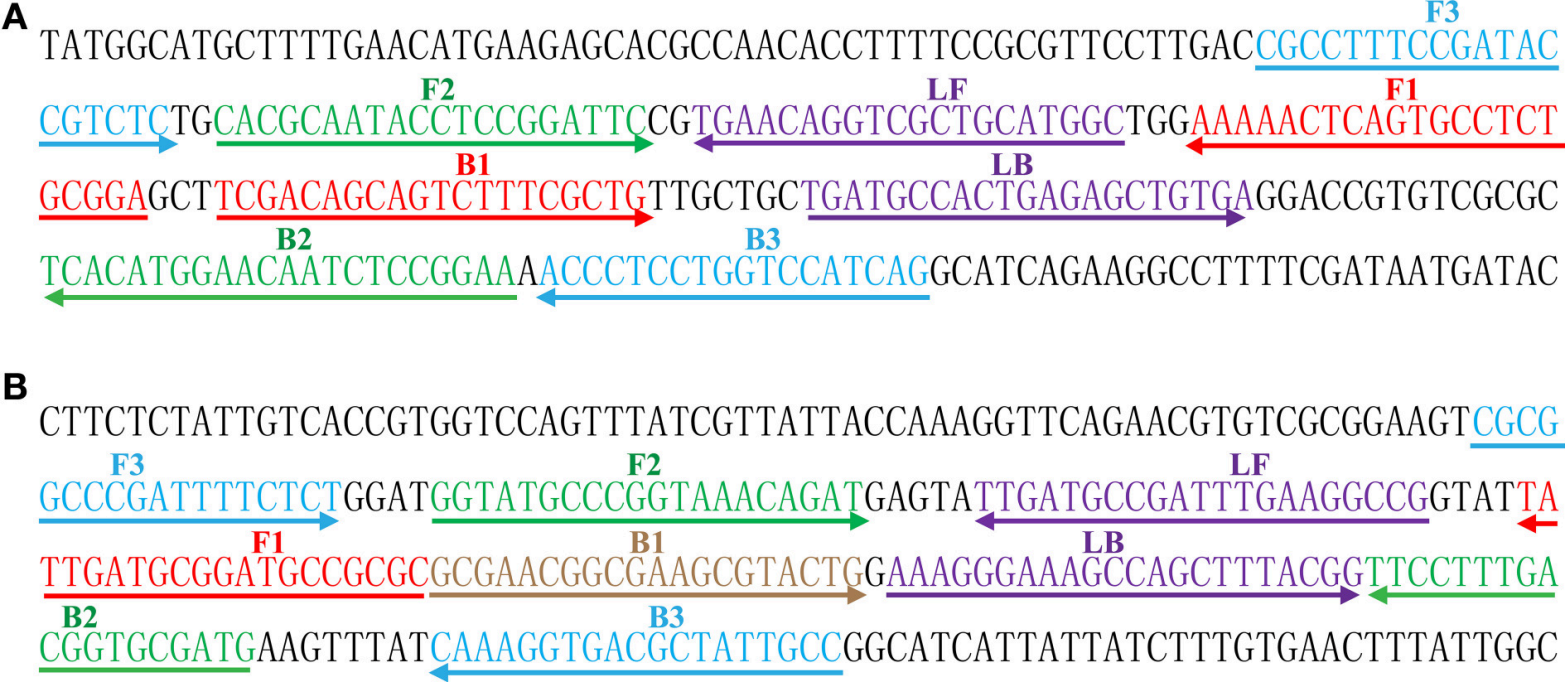
**Sequence and location of ***Shigella*** (***ipaH***) and ***Salmonella*** (***invA***) genes used to design MERT-LAMP primers**. The nucleotide sequences of the sense strands of *ipaH* **(A)** and *invA* **(B)** are listed. The sites of primer sequences were underlined. Left arrows and right arrows showed complementary and sense sequences that are used.

Based on the *ipaH* gene of *Shigella* spp. and *invA* gene of *Salmonella* spp., two sets of MERT-LAMP primers were designed by PrimerExplorer V4 (Eiken Chemical) according to the mechanism of MERT-LAMP technology. Blast analysis confirmed that two sets of MERT-LAMP primers were specific for *Shigella* and *Salmonella*. The dark quenchers used were Black Hole Quencher-1 and Black Hole Quencher-2, and the fluorophores used were HEX and Cy5, which can be monitored in real-time system that was used for carrying out the MERT-LAMP amplifications. All of the oligomers were synthesized and purified by Tian-Yihuiyuan (Beijing, China). The details of target sequences, primer design, primers sequences and locations were shown in Table 1 and Figure 1.

**Table 1:** **The primers used in this study**.

**Primers name^a^**	**Sequences and modifications**	**Length**	**Genes**
*Sal*-F3	5′-CGCGGCCCGATTTTCTCT-3′	18 nt	*invA*
*Sal*-B3	5′-GGCAATAGCGTCACCTTTG-3′	19 nt	
*Sal*-FIP	5′-GCGCGGCATCCGCATCAATAGGTATGCCCGGTAAACAGAT-3′	40 mer	
*Sal*-BIP	5′-GCGAACGGCGAAGCGTACTGCATCGCACCGTCAAAGGAA-3′	39 mer	
*Sal*-EFIP	5′-HEX-TGCAATG-GCGCGGCAT(BHQ-1)CCGCATCAATAGGTATGCCCGGTAAACAGAT-3′	46 mer	
*Sal*-LF	5′-CGGCCTTCAAATCGGCATCAA-3′	21 nt	
*Sal*-LB	5′-AAAGGGAAAGCCAGCTTTACGG-3′	22 nt	
*Shi*-F3	5′-CGCCTTTCCGATACCGTCTC-3′	20 nt	*ipaH*
*Shi*-B3	5′-CTGATGGACCAGGAGGGT-3′	18 nt	
*Shi*-FIP	5′-TCCGCAGAGGCACTGAGTTTTTCACGCAATACCTCCGGATTC-3′	42 mer	
*Shi*-EFIP	5′-Cy5-TGCAATG-TCCGCAGAGGCACT(BHQ-2)GAGTTTTTCACGCAATACCTCCGGATTC-3′	49 mer	
*Shi*-BIP	5′-TCGACAGCAGTCTTTCGCTGTTCCGGAGATTGTTCCATGTGA-3′	42 mer	
*Shi*-LF	5′-GCCATGCAGCGACCTGTTCA-3′	20 nt	
*Shi*-LB	5′-TGATGCCACTGAGAGCTGTGA-3′	21 nt	

a *Sal, salmonella; Shi, Shigella; F, forward; B, backward; FIP, forward inner primer; EFIP, the novel forward inner primer; BIP, backward inner primer; LF, loop forward primer; LB, loop backward primer*.

### Reagents

The DNA extraction kits (QIAamp DNA Mini Kits) were purchased from Qiagen (Beijing, China), and the Nb.*BsrDI* was purchased from New England Biolabs (Beijing, China). The Loopamp kits and Loopamp™ Fluorescent Detection Reagent (FD) were purchase from Eiken Chemical (Tokyo, Japan, and Beijing, China).

### Bacterial strains

A total of 54 strains used in this study were shown in Table 2. All strains were stored in 10% (w/v) glycerol broth an −70°C. These strains were refreshed three times on nutrient agar plate at 37°C, and then were used for enrichment and genomic DNA extraction.

**Table 2:** **Bacterial strains used in this study**.

**Bacteria**	**Serovar/Species**	**Strain no.(source of strain)^a^**	**No. of strains**
*Shigella flexneri*	1d	ICDC-NPS001	1
	4a	ICDC-NPS002	1
	5a	ICDC-NPS003	1
	2b	ICDC-NPS004	1
	1b	ICDC-NPS005	1
	3a	ICDC-NPS006	1
	4av	ICDC-NPS007	1
	3b	ICDC-NPS008	1
	5b	ICDC-NPS009	1
	Y	ICDC-NPS0010	1
	Yv	ICDC-NPS0011	1
	Y	ICDC-NPS0012	1
	X	ICDC-NPS0013	1
	Xv	ICDC-NPS0014	1
*Shigella sonneri*	U	Isolated strains (ICDC)	2
*Shigella dysenteriae*	U	Isolated strains (ICDC)	2
*Shigella boydii*	U	Isolated strains (ICDC)	1
*Salmonella*	Choleraesuis	ICDC-NPSa001	1
	Dublin	ICDC-NPSa002	1
	Enteritidis	ICDC-NPSa003	4
	Typhimurium	ICDC-NPSa004	2
	Weltevreden	ICDC-NPSa005	1
	U	Isolated strains (ICDC)	6
*Listeria monocytogenes*	1/2a	EGD-e	1
	4a	ATCC19114	1
*Enteropathogenic E. coli*	U	Isolated strains (ICDC)	1
*Enterotoxigenic E. coli*	U	Isolated strains (ICDC)	1
*Enteroaggregative E. coli*	U	Isolated strains (ICDC)	1
*Enteroaggregative E. coli*	U	Isolated strains (ICDC)	1
*Enteroinvasive E. coli*	U	Isolated strains (ICDC)	1
*Enterohemorrhagic E. coli*	U	EDL933	1
*Plesiomonas shigelloides*	U	ATCC51903	1
*Enterobacter cloacae*	U	Isolated strains (ICDC)	1
*Enterococcus faecalis*	U	ATCC35667	1
*Yersinia enterocolitica*	U	ATCC23715	1
*Bntorobater sakazakii*	U	Isolated strains (ICDC)	1
*Vibrio cholerae*	U	Isolated strains (ICDC)	1
*Vibrio parahaemolyticus*	U	Isolated strains (ICDC)	1
*Vibrio vulnificus*	U	Isolated strains (ICDC)	1
*Staphylococcus aureus*	U	Isolated strains (ICDC)	1
*Campylobacter jejuni*	U	ATCC33291	1
*Pseudomonas aeruginosa*	U	Isolated strains (ICDC)	1
*Bacillus cereus*	U	Isolated strains (ICDC)	1

a *U, unidentified serotype; ATCC, American Type Culture Collection; ICDC, National Institute for Communicable Disease Control Disease Control and Prevention, Chinese Center for Disease Control and Prevention*.

### Genomic DNA extraction

According to the manufacturer's instructions, the genomic DNA templates were extracted from all culture strains using DNA extraction kits (QIAamp DNA minikits; Qiagen, Hilden, Germany). The extracted templates were tested with ultraviolet spectrophotometer at A260/280 and stored under at −20°C before they were used.

### The normal LAMP assay

To test the usability of two sets of LAMP primers, the LAMP approach either for *Shigella* strains or *Salmonella* strains was performed as the following description. In brief, the reaction was conducted with the Loopamp Kit in a final volume of 25 μl containing 1.6 μM each FIP and BIP primers, 0.8 μM each LF and LB primers, 0.4 μM each F3 and B3 primers, 12.5 μl 2 × reaction mix, 1 μl of *Bst* DNA polymerase (8 U), 1 μl FD, and 1 μl DNA template.

The reaction mixtures of normal LAMP were heated for 1 h at 63°C and then at 85°C for 5 min to stop the reaction. Three mainstream techniques were application to monitor the normal LAMP amplification. The positive amplifications could be directly observed color change by FD reagent, and the products were also detected by electrophoresis on 2% agarose gels with ethidium bromide staining. Moreover, real-time monitoring of normal LAMP reactions was conducted by recording the optical density (OD) at 650 nm every 6 s using the Loopamp Real-time Turbidimeter LA-320C (Eiken Chemical Co., Ltd, Japan). A positive result was defined as a threshold value of >0.1 within 60 min and analysis of each sample (dilution) was determined at least two times.

### The standard MERT-LAMP assay

To evaluate the feasibility of two sets of MERT-LAMP primers, the amplification mixtures of MERT-LAMP were carried out in a final volume of 25 μl containing 0.8 μM EFIP and FIP primers, 1.6 μM BIP primers, 0.8 μM each LF and LB primers, 0.4 μM each F3 and B3 primers, 12.5 μl 2 × reaction mix, 1 μl (8 U) of *Bst* DNA polymerase, 1.5 μl (15 U) of Nb.*BsrDI* endonuclease, and 1 μl DNA template. The mixture was incubated at 63°C for 55 min and then at 85°C for 5 min to stop the amplification. Mixture without DNA template was used as a negative control. After amplification, the MERT-LAMP products were directly observed the color change by FD reagent or confirmed by electrophoresis on 2% agarose gels with ethidium bromide staining. Furthermore, the MERT-LAMP reactions were measured by real-time detection.

### The multiplex MERT-LAMP reaction

For multiplex reactions, the MERT-LAMP approach was conducted as the following system: 25 μl containing 0.4 μM *Shi*-EFIP and *Shi*-FIP primers, 0.8 μM *Shi*-BIP primers, 0.4 μM each *Shi*-LF and *Shi*-LB primers, 0.4 μM each *Shi*-F3 and *Shi*-B3 primers, 0.6 μM *Sal*-EFIP and *Sal*-FIP primers, 1.2 μM *Sal*-BIP primers, 0.6 μM each *Sal*-LF and *Sal*-LB primers, 0.4 μM each *Sal*-F3 and *Sal*-B3 primers, 12.5 μl 2 × reaction mix, 1 μl (8 U) of *Bst* DNA polymerase, 1.5 μl (15 U) of Nb.*BsrDI* endonuclease and 1 μl DNA template DNA each of *Shigella* strains and *Salmonella* strains. The MERT-LAMP mixtures were carried out at 63°C for 55 min in a real-time system, and reaction mixtures without the DNA template were used as a negative control. The lowest detectable template amount were examined in triplicate

In order to determine the optimal amplification temperature of two sets of MERT-LAMP primes, the amplification mixtures of multiplex MERT-LAMP were heated at a constant temperature ranging from 60°C to 67°C for 55 min and then heated at 85°C for 5 min to stop the reaction. Mixtures without DNA template were used as a negative control.

### Evaluation of the sensitivity of the MERT-LAMP assay

To make a comparative analysis of MERT-LAMP, LAMP, qPCR, and PCR assays by using pure culture, genomic DNA templates were serially diluted. The limit of detection (LoD) of MERT-LAMP, LAMP, qPCR, and PCR assays was confirmed by genomic DNA amount of the template. The *Shigella*-qPCR, *Shigella*-PCR, *Salmonella*-qPCR and *Salmonella*-PCR assays have been develop in previous studies, which were employed to verified the LoD of qPCR and PCR technologies (Novinscak et al., 2007; Ueda and Kuwabara, 2009; Mokhtari et al., 2013).

### Evaluation of the specificity of the MERT-LAMP assay

To assess the specificity of the MERT-LAMP methodology, the multiplex MERT-LAMP reactions were performed under the conditions described above with the genomic DNA templates from 55 strains (Table 2). Analysis of each sample was carried out twice independently.

### Practical application of MERT-LAMP to *Shigella* and *Salmonella* detection in food samples

In order to test the applicability of MERT-LAMP technology in food sample, *Shigella flexneri* serotype 1d (ICDC-NPS001) and *Salmonella Enteritidis* (ICDC-NPSa003) were simultaneously added to the pasteurized milk samples, which were purchased from a grocery store in Beijing. The milk samples were confirmed as being *Shigella*- and *Salmonella*-negative by traditional culture assay and PCR. Firstly, to test the minimal detectable colony forming units (CFUs), the cultures with *Shigella flexneri* or *Salmonella Enteritidis* strains were serially diluted (10^−1^–10^−9^), and the aliquots of 100 μl appropriate dilution (10^−6^) was plated in triplicate on brain heart infusion (BHI). The CFUs were counted after 24 h at 37°C. The following steps, the aliquots of 100 μl appropriate dilutions with *Shigella flexneri* and *Salmonella Enteritidis* was simultaneously inoculated into the milk samples, and the number of *Shigella* was adjusted to approximate 2.9 × 10^5^, 2.9 × 10^4^, 2.9 × 10^3^, 2.9 × 10^2^, 2.9 × 10^1^, and 2.9 × 10^0^ CFU/ml, *Salmonella* for 3.2 × 10^5^, 3.2 × 10^4^, 3.2 × 10^3^, 3.2 × 10^2^, 3.2 × 10^1^, and 3.2 × 10^0^ CFU/ml. Simultaneously, aliquots (100 μl) of the artificially contaminated milk was used for DNA extraction, and the supernatants (2 μl) were used for multiplex MERT-LAMP, LAMP, real-time PCR and PCR detection. Non-contaminated milk sample was selected as negative control. This performance was carried out in triplicate independently.

## Results

### Confirmation of *Shigella*- and *Salmonella*-LAMP products

**Figure 2:**
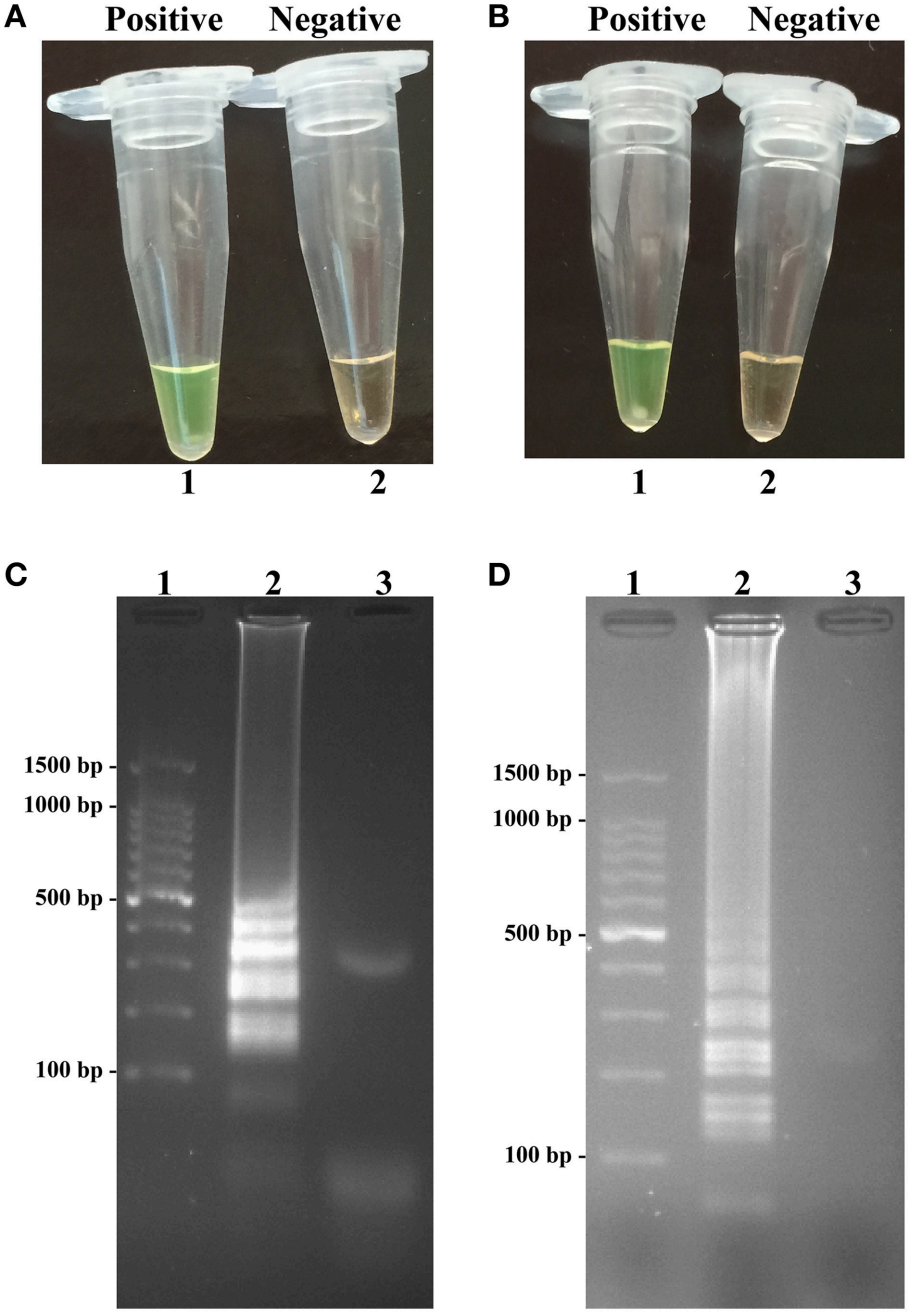
**Confirmation and detection of ***Shigella***- and ***Salmonella***-LAMP products. (A,B)** Color change of *Shigella*- and *Salmonella*-LAMP tubes; tube 1, positive amplification; tube 2, negative amplification. **(C,D)**, 2% agarose gel electrophoresis applied to *Shigella*- and *Salmonella*-LAMP products; lane 1, DL 100-bp DNA marker; lane 2, positive LAMP reaction, lane 3, negative LAMP reaction.

The color change of positive amplifications in *Shigella*- and *Salmonella*-LAMP tubes from light gray to green were directly observed by naked eyes within 1 h incubation periods (Figures 2A,B). The normal LAMP products were also detected by 2% agarose gel electrophoresis, and the typical ladder-like patterns were visual (Figures 2C,D). Thus, two sets of normal LAMP primers for *Shigella* and *Salmonella* detection were good candidates for establishing the MERT-LAMP methodologies.

### Sensitivity of *Shigella*- and *Salmonella*-LAMP assay in pure culture

**Figure 3:**
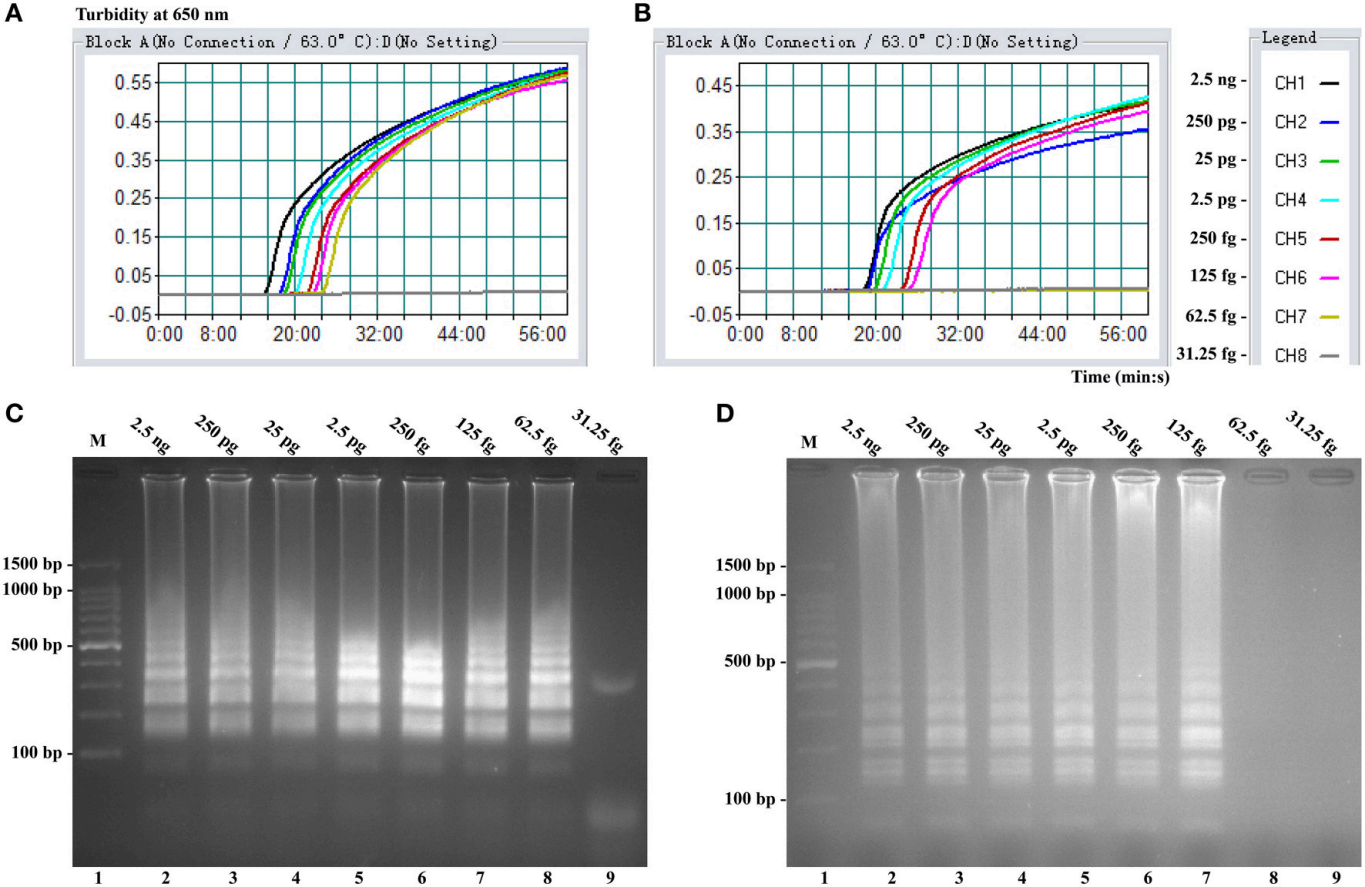
**Sensitivity of the ***ipaH***- and ***invA***-LAMP assays using serially genomic DNA with ***Shigella*** strains and ***Salmonella*** strains as templates**. Sensitivity of *ipaH*-LAMP **(A)** and *invA*-LAMP **(B)** for *Shigella* and *Salmonella* detection was analyzed by real-time measurement of turbidity and the corresponding curves of concentrations of genomic DNA were marked in the figure. The LoD of *ipaH*-LAMP assay was 62.5 fg per tube, and the *invA*-LAMP for 125 fg per reaction. Sensitivity of *ipaH*-LAMP **(C)** and *invA*-LAMP **(D)** for *Shigella* and *Salmonella* detection was monitored by 2% agarose gel electrophoresis, and the positive amplifications were seen as a ladder-like pattern on 2% agarose gel electrophoresis analysis. Lane 1, DL 100-bp DNA marker.

Sensitivity of LAMP reactions on *Shigella* and *Salmonella* were examined by analyzing products generated from the serial dilutions (2.5 ng, 250 pg, 25 pg, 2.5 pg, 250 fg, 125 fg, 62.5 fg, and 31.25 fg per microliter) of the *Shigella* and *Salmonella* genomic DNA templates in triplicate. As shown in Figures 3A,B, the LAMP amplifications were monitored by real-time turbidity detection; the LoD of *Shigella*-LAMP approaches was 62.5 fg/reaction (Figure 3A), and the LoD of *Salmonella*-LAMP assays was 125 fg/reaction (Figure 3B). Moreover, the positive reactions by 2% agarose gel electrophoresis were observed as a ladder-like pattern, the LoD of the agarose gel electrophoresis detection for *Shigella*- and *Salmonella*-LAMP reactions was identical with turbidity measurement (Figures 3C,D).

### Confirmation and detection of MERT-LAMP products in nonreal-time format

**Figure 4:**
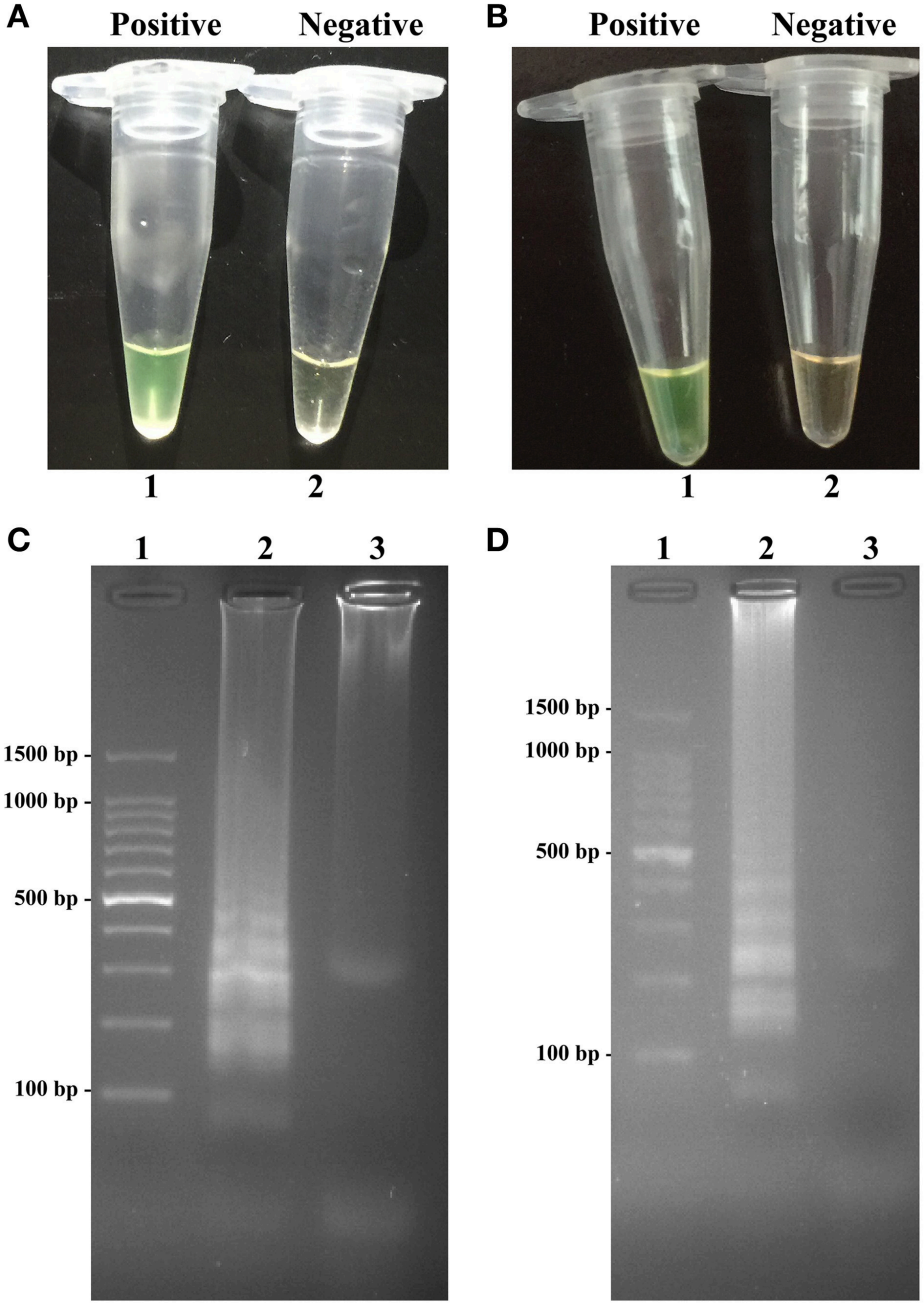
**Confirmation and detection of ***ipaH***- and ***invA***-MERT-LAMP products. (A,B)** Color change of *ipaH*- and *invA*-MERT-LAMP tubes; tube 1, positive amplification; tube 2, negative amplification. **(C,D)** 2% agarose gel electrophoresis applied to *ipaH*- and *invA*-MERT-LAMP products; lane 1, DL 100-bp DNA marker; lane 2, positive LAMP reaction, lane 3, negative LAMP reaction.

To validate the utility of two sets of MERT-LAMP primers, reaction systems were conducted in the presence or absence genomic DNA templates according to the standard MERT-LAMP condition. The color change of positive MERT-LAMP reactions in *ipaH*- and *invA*-MERT-LAMP tubes from light gray to green were directly seen by naked eyes within 1 h incubation periods (Figures 4A,B). Furthermore, the positive products were also detected by 2% agarose gel electrophoresis, and the typical ladder-like patterns were visible but not in negative control (Figures 4C,D). The results indicated the *ipaH*- and *invA*-MERT-LAMP primers were feasible for *Shigella* and *Salmonella* detection.

### Analytical sensitivity of MERT-LAMP detection for a single target

**Figure 5:**
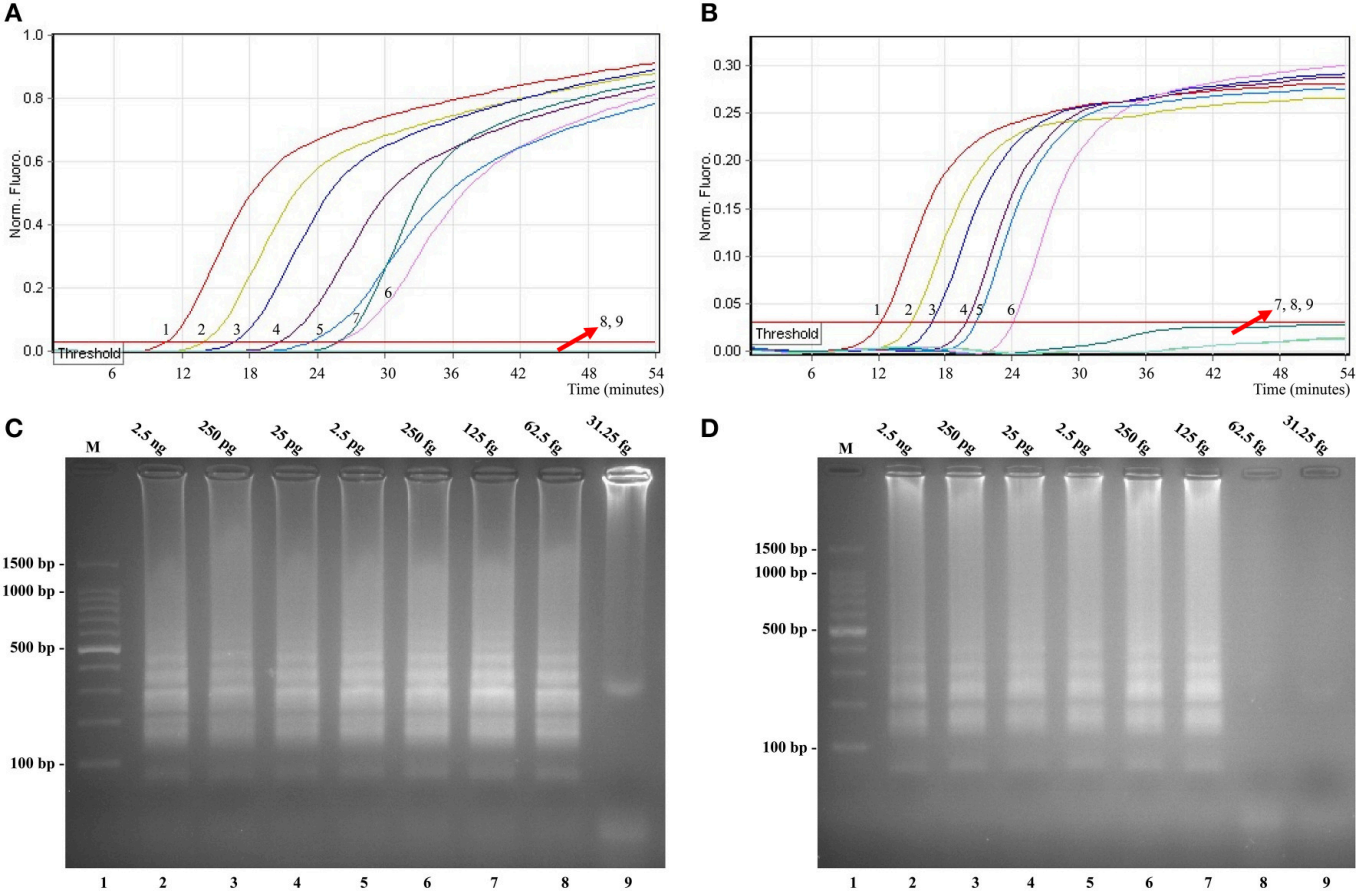
**Sensitivity of the ***ipaH***- and ***invA***-MERT-LAMP assays using serially genomic DNA with ***Shigella*** strains and ***Salmonella*** strains as templates**. Sensitivity of *ipaH*-MERT-LAMP **(A)** and *invA*-MERT-LAMP **(B)** for *Shigella* and *Salmonella* detection was monitored by real-time format, and signals 1, 2, 3, 4, 5, 6, 7, 8, and 9 represent DNA levels of 2.5 ng, 250 pg, 25 pg, 2.5 pg, 250 fg, 125 fg, 62.5 fg, and 31.25 fg per vessel and negative control. The LoD of *ipaH*-MERT-LAMP assay was 62.5 fg per tube, and the *invA*-MERT-LAMP for 125 fg per reaction. Sensitivity of *ipaH*-MERT-LAMP **(C)** and *invA*-MERT-LAMP **(D)** for *Shigella* and *Salmonella* detection was analyzed by 2% agarose gel electrophoresis, and the positive amplifications were observed as a ladder-like pattern on 2% agarose gel electrophoresis analysis. Lane 1, DL 100-bp DNA marker.

In this study, we evaluated MERT-LAMP amplification in a single target format by using separate detection of *ipaH* (*Shigella* spp.-specific gene) and *invA* (*Salmonella* spp.-specific gene) from an *Shigella* and *Salmonella* genomic DNA templates with EFIP in each reaction. As expected, the release of quenching was observes as a robust increase of Cy5 and Hex signals, and positive results were obtained in ~12 min (Figures 5A,B). The LoD of MERT-LAMP methodology for independently detecting *ipaH* and *invA* genes was 62.5 and 125 fg of genomic DNA templates per reaction, respectively (Figures 5A,B). Furthermore, the positive amplifications by 2% agarose gel electrophoresis were seen as the typical ladder-like patterns were visible but not in negative control, and the LoD of the agarose gel electrophoresis detection for *Shigella*- and *Salmonella*-LAMP amplifications was identical with real-time measurement (Figures 5C,D).

The LoD of MERT-LAMP, LAMP, qPCR, and PCR assays on *Shigella* was 62.5 fg, 62.5 fg, 2.5 pg and 25 pg, and on *Salmonella* was 125 fg, 125 fg, 2.5 pg, and 25 pg, respectively (Table 3, Figures 3, 5). The results indicated that the sensitivity of MERT-LAMP technique for detecting a single target was identical with normal LAMP approach, whereas was at least 10- and 100-fold more sensitive than that of qPCR and PCR assays, respectively.

**Table 3:** **The LoD and time for single MERT-LAMP targeting ***ipaH*** and ***invA*** genes compared with that of qPCR and conventional PCR approaches**.

**Assays**	**Isothermal amplification**	**Regions recognized**	**Multiplex detection**	**LoD for *Shigella* spp./*Salmonella* spp. (no./reaction)**	**Fastest time (minutes)**	**LoD time (minutes)^a^**
MERT-LAMP	+	8	+	62.5 fg/125 fg	12	26
LAMP	+	8	–	62.5 fg/125 fg	18	28
qPCR	–	3	+	2.5 pg/2.5 pg	32	56
PCR	–	2	+	25 pg/25 pg	150	150

a *The LoD values are the lowest gnomic DNA level that was positively amplified in triplicate. The positive results of qPCR were obtained as c_t_ values, which converted to time for detection*.

*MERT-LMAP, multiple endonuclease restriction real-time loop-mediated isothermal amplification, qPCR, quantitative real-time PCR, LoD, limit of detection*.

### The optimal amplification temperature of *ipaH*- and *invA*-MERT-LAMP assays

**Figure 6:**
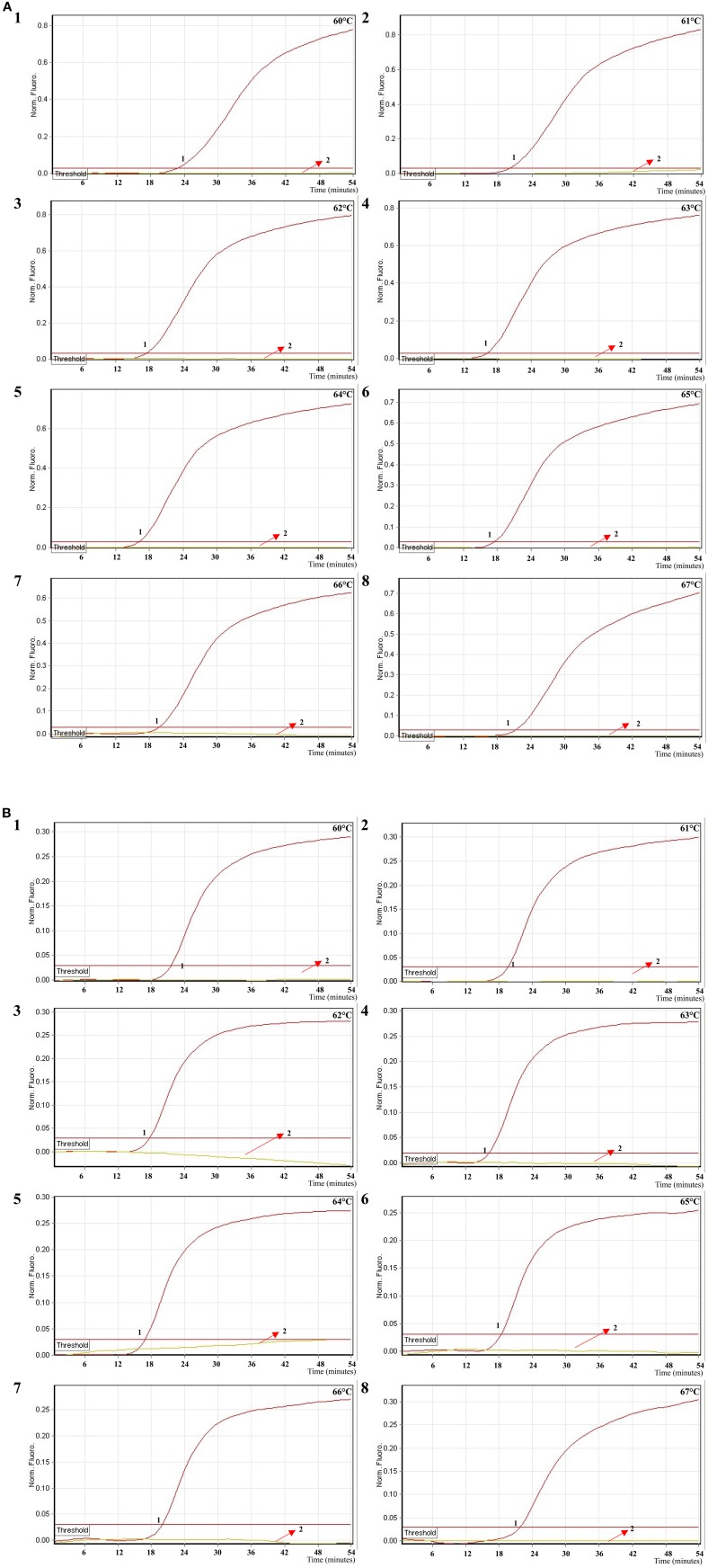
**The optimal temperature for multiplex MERT-LAMP assay**. Two sets of MERT-LMAP primers targeting *ipaH* and *invA* genes were used in the same reaction tube, **(A,B)** were simultaneously obtained from Cy5 (labeling *Shi*-EFIP of *ipaH*) and Hex (labeling *Sal*-EFIP of *invA*) channels, respectively. The multiplex MERT-LAMP amplifications were detected by means of real-time format, and the corresponding curves of DNA concentrations were listed. Signal 1 indicates *Shigella flexneri* strains of in Cy5 channel **(A)**, *Salmonella Enteritidis* strain for Hex channel **(B)**, and signal 2 indicates negative control. Eight kinetic graphs (1–8) were generated at different amplification temperature (60°C–67°C) with *Shigella* genomic DNA at the level of 2.5 ng in Cy5 channel **(A)**; another eight kinetic graphs (1–8) were yielded at different detection temperature (60°C–67°C) with *Salmonella* genomic DNA at the level of 2.5 ng in Hex channel **(B)**. The graphs from 62°C to 65°C show robust amplification.

In order to examine the optimal detection temperature of multiplex MERT-LMAP approach, the multiplex MERT-LMAP amplifications, which simultaneously contained the *Shigella flexneri* and *Salmonella enteritidis* genomic DNA templates at the level of 2.5 ng per tube, were performed at distinct temperatures (60°C–67°C) according to the multiplex MERT-LAMP reaction. The results were analyzed by means of real-time format, and the typical kinetics graphs were produced (Figure 6). Each detection temperature generated a robust signal corresponding to Cy5 and Hex channel, with the faster amplification observed for assay temperature of 62°C–65°C, which were considered as the standard temperature for multiplex MERT-LAMP detection. The detection temperature of 63°C was used for the rest of multiplex MERT-LAMP reaction conducted in this study.

### Analytical sensitivity of MERT-LAMP detection for multiple targets in a reaction

**Figure 7:**
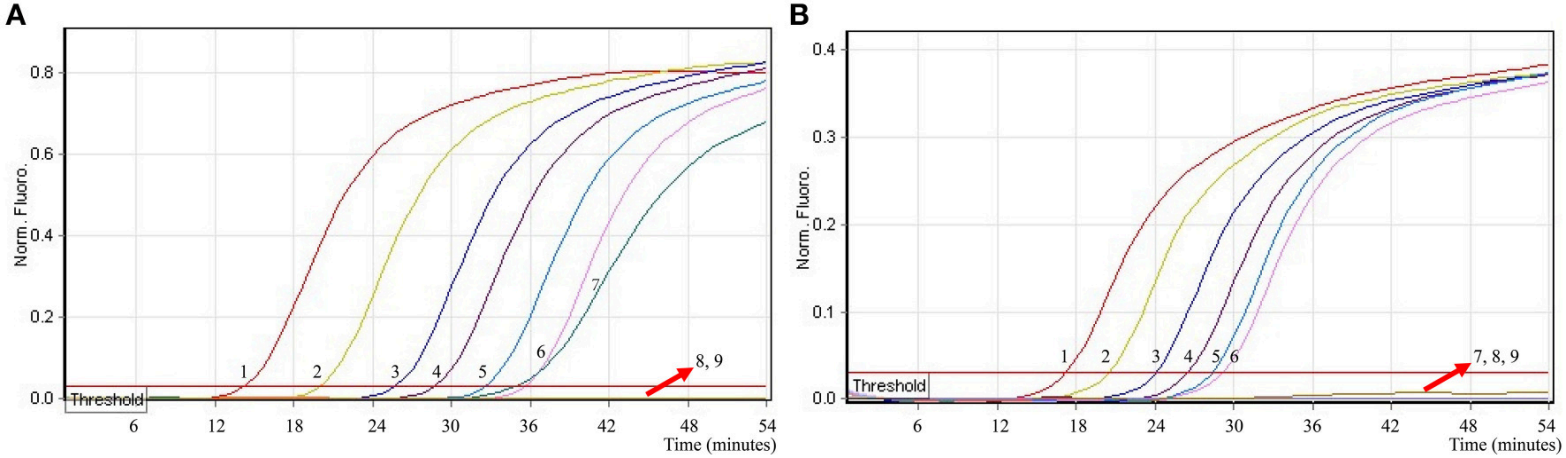
**The sensitivity of multiplex MERT-LAMP assay for simultaneously detecting two target pathogens**. Two sets of MERT-LMAP primers targeting *ipaH* and *invA* genes were simultaneously added to a reaction tube. **(A,B)** were simultaneously generated from Cy5 (labeling *Shi*-EFIP of *ipaH*) and Hex (labeling *Sal*-EFIP of *invA*) channels, respectively. Sensitivity of multiplex-MERT-LAMP for simultaneously detecting *Shigella* **(A)** and *Salmonella* **(B)** was monitored by real-time format, and signals 1, 2, 3, 4, 5, 6, 7, 8, and 9 represent DNA levels of 2.5 ng, 250 pg, 25 pg, 2.5 pg, 250 fg, 125 fg, 62.5 fg, and 31.25 fg per vessel and negative control. The LoD of multiplex MERT-LAMP assay for *Shiglla* detection was 62.5 fg per tube, and the LoD of multiplex MERT-LAMP for *Salmonella* detection was 125 fg per reaction.

In order to simultaneously identify multiple targets in a single reaction, we slightly modified the amount of the primers on the base of standard MERT-LAMP reaction, and the multiplex MERT-LAMP systems were also performed at 63°C for 55 min. Two different fluorescence curves were simultaneously yielded from multiplex MERT-LAMP reactions containing two complete primer sets and their corresponding genomic DNA templates (Figure 7). The MERT-LAMP assay successfully detected *Shigella* and *Salmonella* in a single reaction, and simultaneously presented two sets of robust signals for two targets. The LoD of MERT-LAMP technology for simultaneously differentiating *ipaH* and *invA* genes was 62.5 and 125 fg of each genomic DAN per tube, respectively (Figure 7). No difference of sensitivity was seen between detecting multiple targets and a single target in a MERT-LAMP system.

### Analytical specificity of the multiplex MERT-LAMP methodology

**Figure 8:**
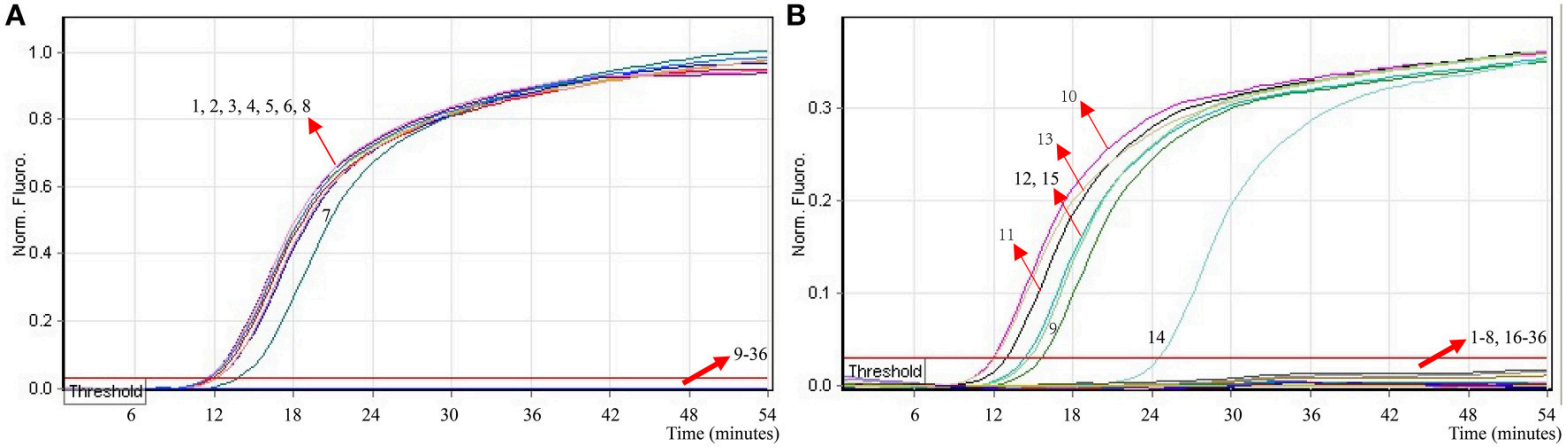
**The specificity of multiplex MERT-LMAP detection for different strains**. The multiplex MERT-LAMP reactions were conducted using different genomic DNA templates and were analyzed by means of real-time format. **(A,B)** were simultaneously yielded from Cy5 and Hex channels. Signals 1–8, *Shigella flexneri* strains of serovar 1d (ICDC-NPS001), 4a (ICDC-NPS002), 5a (ICDC-NPS003), 2b (ICDC-NPS004), 1b (ICDC-NPS005), *Shigella boydii, Shigella sonneri* and *Shigella dysenteriae*; signals 9–13, *Salmonella Choleraesuis* (ICDC-NPSa001), *Salmonella Dublin* (ICDC-NPSa002), *Salmonella Enteritidis* (ICDC-NPSa003), *Salmonella Typhimurium* (ICDC-NPSa004), *Salmonella Weltevreden* (ICDC-NPSa005); signals 14 and 15, two *Salmonella* strains of unidentified serotype; signals 16–35, *Listeria monocytogenes* stains of serovar 1/2a (EGD-e), *Listeria monocytogenes* stains of serovar 4a (ATCC19114), *Enteropathogenic E. coli, Enterotoxigenic E. coli, Enteroaggregative E. coli, Enteroaggregative E. coli, Enteroinvasive E. coli, Enterohemorrhagic E. coli, Plesiomonas shigelloides, Enterobacter cloacae, Enterococcus faecalis, Yersinia enterocolitica, Bntorobater sakazakii, Vibrio cholerae, Vibrio parahaemolyticus, Vibrio vulnificus, Staphylococcus aureus, Campylobacter jejuni, Pseudomonas aeruginosa*, and *Bacillus cereus*, signal 36, negative control.

To evaluate the specificity of multiplex MERT-LAMP technique, a total of 54 strains, including 19 *Shigella* and 15 *Salmonella* and 20 non-*Shigella* and non-*Salmonella*, were subjected to MERT-LAMP reaction. By observation, positive results could be generated only when genomic DNAs of *Shigella* strains and *Salmonella* strains were used as templates in multiplex MERT-LAMP assay, and the target pathogens were correctly distinguished (Figure 8). In contrast, negative control, non-*Shigella* stains and non-*Salmonella* strains tested by multiplex MERT-LAMP technology were negative after 55-min incubation period. These results showed that the multiplex MERT-LAMP assay presented here was specific to target sequence identification.

### Multiplex MERT-LAMP technology for artificially contaminated milk sample

**Figure 9:**
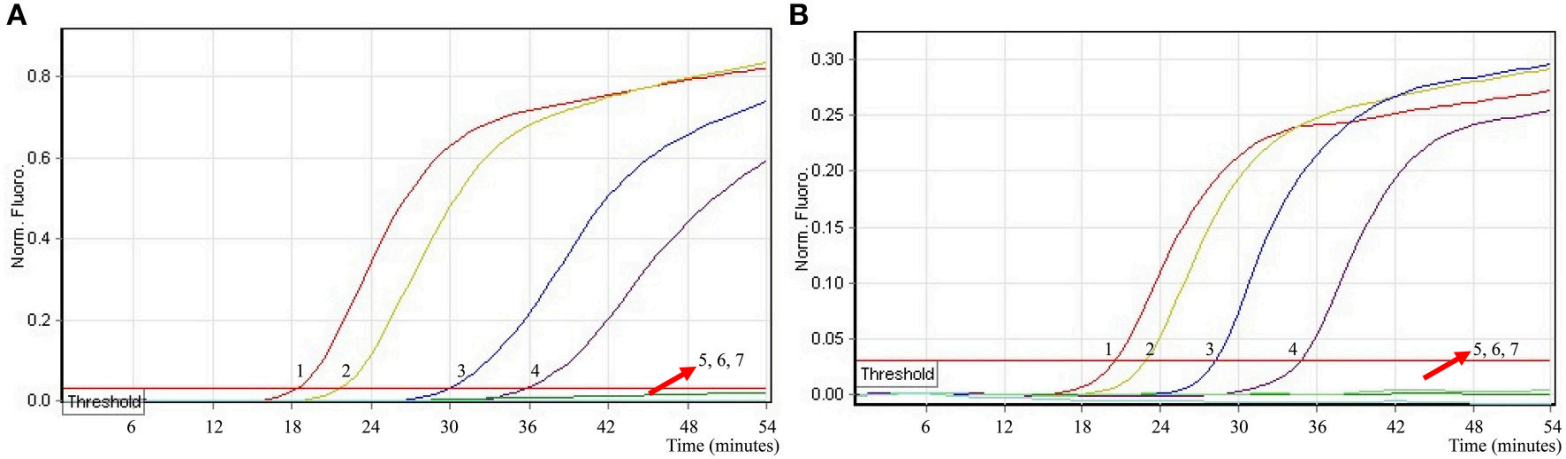
**The sensitivity of multiplex MERT-LAMP assay for simultaneously detecting two target pathogens in artificially contaminated milk samples**. Two sets of MERT-LMAP primers targeting *ipaH* and *invA* genes were added to a reaction tube. **(A,B)** were simultaneously generated from Cy5 (labeling *Shi*-EFIP of *ipaH*) and Hex (labeling *Sal*-EFIP of *invA*) channels, respectively. Sensitivity of multiplex-MERT-LAMP for simultaneously detecting *Shigella* **(A)** and *Salmonella* **(B)** in artificially contaminates milk samples was analyzed by real-time format, and signals 1, 2, 3, 4, 5, 6, and 7 represent *Shigella* DNA levels of 5800, 580, 58, 5.8, 0.58, and 0.058 CFU per reaction and negative control; *Salmonella* DNA levels for 6400, 640, 64, 6.4, 0.64, and 0.064 CFU per reaction and negative control. The LoD of multiplex MERT-LAMP assay for *Shiglla* detection in artificially contaminates milk samples was 5.8 CFU per tube, and the LoD of multiplex MERT-LAMP for *Salmonella* detection in artificially contaminates milk samples was 6.4 CFU per reaction.

To ascertain the applicability the MERT-LAMP technology as a surveillance tool for *Shigella* and *Salmonella* in food, the MERT-LAMP assay was tested by the artificially inoculating *Shigella* stain and *Salmonella* strain into milk sample. The multiplex MERT-LAMP approach could yield positive amplification when the contaminated numbers of *Shigella* and *Salmonella* were more than 2.9 × 10^2^ CFU/ml (~5.8 CFU/tube) and 3.2 × 10^2^ CFU/ml (~6.4 CFU/tube), respectively, and the two target pathogens were simultaneously detected in a single MERT-LAMP reaction (Figure 9, Table 4). The non-contaminated milk sample was found to be negative.

**Table 4:** **Comparison of MERT-LMAP, LAMP, qPCR, and PCR assay for detection of ***Shigella*** spp. and ***Salmonella*** spp. in artificially contaminated milk samples**.

**Detection methods^a^**	**Multiplex detection**	**LoD (no./reaction)**	
		***Shigella* spp. detection**	***Salmonella* spp. detection**
MERT-LAMP	+	5.8 CFU ~ (2.7 × 10^2^ CFU/ml)	6.4 CFU ~ (3.2 × 10^2^ CFU/ml)
LAMP	–	5.8 CFU ~ (2.7 × 10^2^ CFU/ml)	6.4 CFU ~ (3.2 × 10^2^ CFU/ml)
qPCR	–	58 CFU ~ (2.7 × 10^3^ CFU/ml)	64 CFU ~ (3.2 × 10^3^ CFU/ml)
PCR	–	580 CFU ~ (2.7 × 10^4^ CFU/ml)	640 CFU ~ (3.2 × 10^4^ CFU/ml)

a *MERT-LAMP, multiple endonuclease restriction real-time loop-mediated isothermal amplification; LAMP, loop-mediated isothermal amplification*.

The detection limit of multiplex MERT-LAMP technology was consistent with that of normal LAMP detection only for *Shigella* or *Salmonella*, respectively (Table 4). Comparatively, the qPCR and PCR assays produced positive results when the contaminate numbers of *Shigella* amounted to more than 2.9 × 10^3^ CFU/ml (~58 CFU/tube) and 2.9 × 10^4^ CFU/ml (~580 CFU/tube), *Salmonella* for 3.2 × 10^3^ CFU/ml (~64 CFU/tube), and 3.2 × 10^4^ CFU/ml (~640 CFU/tube), respectively. The results revealed that the sensitivity of multiplex MERT-LAMP assays was 10- and 100-fold more sensitive than that of qPCR and PCR methodologies (Table 4).

## Discussion

Previous studies have reported that *Shigella* and *Salmonella* could contaminate similar types of samples, thus it would be significant to simultaneously identify the two pathogens (Li and Mustapha, 2004; MacRitchie et al., 2014). Culture-based techniques and biochemical are the most common detection assays, while require a long enrichment time and subsequent identification (Mokhtari et al., 2012; Soria et al., 2013). PCR-based technologies have been successfully developed to detect *Shigella* and *Salmonella* from various food products and environment samples (Okamura et al., 2009; Liew et al., 2014). However, these techniques require a sophisticated apparatus to denature the template DNA, which significantly limit its wider application in under-resourced settings and in field laboratories (Wang et al., 2014). In recent years, the LAMP methodology was widely applied to detect *Shigella* and *Salmonella* due to the simplicity and rapidity, while it was restricted to identify only a single target in a vessel.

As a novel isothermal detection technology, MERT-LAMP assay was recently devised and the working principle was successfully validated (Wang et al., 2015a). Under the isothermal condition, the MERT-LAMP methodology permitted real-time detection of multiple targets in a single reaction, and the amplification and detection were conducted simultaneously within 55 min. In this study, the MERT-LAMP technique for the simultaneous differentiation of *Shigella* and *Salmonella* was successfully developed and evaluated. The MERT-LAMP amplifications were carried out at only one isothermal step, obviating the use of a thermal cycling equipment, and did not further pyrosequencing and agarose gel detection. Therefore, the multiplex MERT-LAMP approach significantly reduced reagents, materials and effort used, and identification and amplification were performed at the same time, alleviating the use of further procedures. Moreover, the reaction vessels were kept closed in the whole course of the experiment, which effectively eliminated any carryover contamination.

Two species-specific genes (*ipaH* and *invA*) present in both *Shigella* and *Salmonella* were used as target sequences for designing MERT-LAMP primers (Vantarakis et al., 2000; Hara-Kudo et al., 2005). The specificity of multiplex MERT-LAMP reactions was successful examined, and the positive results were generated in the assay of *Shigella* strains and *Salmonella* strains but not for non-*Shigella* strains and non-*Salmonella* strains. Furthermore, the MERT-LAMP established here can simultaneously detect and correctly distinguish the target pathogens in a sample, which can be completed in only one isothermal detection step with easily interpretable results. Therefore, the MERT-LAMP assay combined with hand-held diagnostic devices can be as a potential tool for detecting target pathogens, which can be widely applied to various fields, such as point-of-care diagnosis, field testing, and more.

The established multiplex MERT-LAMP assay detected as little as 62.5 and 125 fg of *Shigella* and *Salmonella* DNA per tube in the pure cultures, which was consistent with that of normal LAMP and singleplex MERT-LAMP reactions. However, the normal LAMP technology was limited to detect only one target template (Yan et al., 2014). The analytical sensitivity of multiplex MERT-LMAP assay indicated that it could screen marginal amounts of target sequences and was at least 10- and 100-fold more sensitive than that of qPCR and PCR approaches, respectively. With regard to assay speed and sensitivity level obtained in our study, the MERT-LAMP methodology performed better than qPCR and PCR techniques, thus significantly shortening the total detection time (Table 3). Therefore, the MERT-LAMP assay presented here was recommended as a potential diagnostic tool for screening *Shigella* strains and *Salmonella* strains in a variety of samples.

To test the practical application of MERT-LAMP detection of *Shigella* strains and *Salmonella* strains in food products, artificially contaminated milk samples were analyzed by conventional PCR, qPCR, LAMP, and multiplex MERT-LAMP assays. The detection limit of multiplex MERT-LAMP for *Shigella* strains and *Salmonella* strains detection in artificially contaminated milk samples was 5.8 and 6.4 CFU per tube, which was also identical with normal LAMP analysis. The two target pathogens could be simultaneously screened within 40 min with only isothermal incubation step and the positive results were presented in real-time format (Figure 9). Moreover, the LoD of multiplex MERT-LAMP technology for artificially contaminated milk samples was 10- and 100-fold more sensitive than qPCR and conventional PCR assays. Herein, the multiplex MERT-LAMP approach was more suitable than LAMP, qPCR, and PCR techniques for multiplex, rapid, specific and sensitive for detection of *Shigella* strains and *Salmonella* strains in food samples.

In conclusion, a rapid, multiplex, specific, and sensitive MERT-LAMP assay for simultaneous detection of *Shigella* strains and *Salmonella* strains was established by targeting *ipaH* and *invA* genes, and the specificity, sensitivity and practical application were successfully evaluated. Thus, the novel MERT-LAMP assay could be used as a potential tool for facilitating simultaneous monitoring of *Shigella* strains and *Salmonella* strains in variety of samples.

### Conflict of interest statement

The authors declare that the research was conducted in the absence of any commercial or financial relationships that could be construed as a potential conflict of interest.
